# Design and Implementation of an Intrinsically Safe Liquid-Level Sensor Using Coaxial Cable

**DOI:** 10.3390/s150612613

**Published:** 2015-05-28

**Authors:** Baoquan Jin, Xin Liu, Qing Bai, Dong Wang, Yu Wang

**Affiliations:** Key Laboratory of Advanced Transducers and Intelligent Control Systems, Ministry of Education, Taiyuan University of Technology, No.79 Yingzexi Street, Taiyuan 030024, China; E-Mails: liuxintyut0902@163.com (X.L.); baiqingchina@163.com (Q.B.); wangdongwind@gmail.com (D.W.); wangyu@tyut.edu.cn (Y.W.)

**Keywords:** PVC coaxial cable, capacitive liquid-level sensor, intrinsically safe circuit, piecewise linearization

## Abstract

Real-time detection of liquid level in complex environments has always been a knotty issue. In this paper, an intrinsically safe liquid-level sensor system for flammable and explosive environments is designed and implemented. The poly vinyl chloride (PVC) coaxial cable is chosen as the sensing element and the measuring mechanism is analyzed. Then, the capacitance-to-voltage conversion circuit is designed and the expected output signal is achieved by adopting parameter optimization. Furthermore, the experimental platform of the liquid-level sensor system is constructed, which involves the entire process of measuring, converting, filtering, processing, visualizing and communicating. Additionally, the system is designed with characteristics of intrinsic safety by limiting the energy of the circuit to avoid or restrain the thermal effects and sparks. Finally, the approach of the piecewise linearization is adopted in order to improve the measuring accuracy by matching the appropriate calibration points. The test results demonstrate that over the measurement range of 1.0 m, the maximum nonlinearity error is 0.8% full-scale span (FSS), the maximum repeatability error is 0.5% FSS, and the maximum hysteresis error is reduced from 0.7% FSS to 0.5% FSS by applying software compensation algorithms.

## 1. Introduction

Liquid level sensing in flammable and explosive environments has always been a technical challenge. Generally, liquid level can be detected with reported various methods such as the float, ultrasonic [1], magnetostrictive [2], differential pressure [3], optical [4,5], and capacitive methods [6,7], *etc.* The conventional float sensors commonly utilize a bulky float as the measuring element. Although these sensors have a simple structure and a cheaper price, their application is limited because of their vulnerability to mechanical damage and high maintenance cost. Moreover, weight and volume of the mechanical system must also be taken into consideration [8]. Ultrasonic liquid level sensors usually work by emitting high-frequency acoustic signals that are reflected back to and detected by the transducer. The transmission time of the signal corresponds to the liquid level. The ultrasonic sensors possess advantages of simple structure, easy installation and maintenance, but they are susceptible to interferences, and the existence of the RLC resonant circuit with large energy storage components makes them difficult to achieve intrinsically safe performance. The magnetostrictive liquid-level measurement technique is based on the detection of the propagation time of the elastic wave produced by the magnetostrictive effect in ferromagnetic materials. With the advantages of high precision, large-scale and high security, magnetostrictive liquid-level sensor can be used for liquid-level measurement in flammable and explosive environments. However, the magnetostrictive liquid-level sensor suffers from the significant disadvantage of requiring the active float, which is easy to get stuck in special environments such as the turbid liquid. The differential pressure liquid-level sensor, whose static pressure produced by the liquid column corresponds to the liquid level, is the most widely used liquid-level sensors currently due to the characteristics of stable performance, high precision and low cost, *etc.* Nevertheless, the differential pressure liquid-level sensor exhibit some drawbacks such as it is easily jammed or blocked, even results in measuring invalidation in complex environments. Fiber optic liquid-level sensor shows attractive advantages of anti-electromagnetic interference, multiplexing capabilities, fast response, and robustness toward harsh environments [9], and is usually considered to be suitable for liquid-level measurement in flammable and explosive environments, however, they are not without flaws. For example, the fiber optic sensors are unable to measure the turbid liquid level and other liquid with sticky substances, which may adhere to the surface of the sensing probe. Capacitive sensors are increasingly common over other existing ones in the field of electrical liquid-level sensors due to remarkable advantages such as low cost, high linearity, low energy dissipation, and easy adjustability to the geometry of the application [10]. For this reason, capacitive sensors are widely used in the measurement of liquid-level [11,12] since they are found to have very good sensitivity in water [13]. Unfortunately, currently used capacitive liquid level sensors are limited in application to potentially flammable or explosive environments due to the complex structure and non-intrinsic safety. In summary, the states of the art is shown in Table 1.

The measuring environment of the underground coal mine, which is characterized by the presence of explosive gases and particles such as methane and coal dust, is extremely complex. Besides, the measuring liquid is slime water, which contains a lot of coal powder and clay. Therefore, the above liquid-level sensors can hardly reflect real-time hydrological conditions accurately in the underground coal mine due to their own characteristics. Thus, there remains a need for a novel, intrinsically safe liquid level sensor, which is safe, reliable, accurate, low cost, and especially suitable for the underground coalmine. In [14], a multi-sensor system using plastic optical fibers is developed for intrinsically safe level measurements; the linearity is better than 1.5% full-scale span (FFS) and a resolution better than 0.5% FFS is obtained. However, the sensor cannot be applied to measure the turbid liquid level and other liquid with sticky substance, besides, the measurement results should be further improved. In [15], a side-coupled optic-fiber liquid level sensor is designed to achieve intrinsically safe measurement to liquid level in flammable environments. However, there are also problems in this sensor, such as the narrow measuring range (20 cm) and susceptibility to the dip of liquid. In [16], an intrinsically safe intelligent water-level monitor used in coal mine, which converts the pressure signal into frequency signal, is proposed. However, the sensor is easily jammed or blocked in turbid liquid. On the other hand, capacitive sensors have attracted considerable attention in liquid level measurement. Regrettably, currently used capacitive liquid level sensors cannot meet the requirement of liquid-level sensing in flammable and explosive environments. Therefore, once the intrinsic safety issue is resolved, capacitive sensing design reduces the costs and may provide a handy solution for liquid-level measurement in flammable and explosive environments. Generally, intrinsically safe circuit is indispensable for the device installed in the flammable and explosive gas environments. According to IEC 60079-0-2007 Explosive atmospheres—Part 0: Equipment-General requirements [17] and IEC 60079-11-2006 Explosive atmospheres—Part 11: Equipment protection by intrinsic safety “i” [18], intrinsically safe circuit is defined as that it is unable to release sufficient electrical or thermal energy to cause an ignition of a flammable mixture in case of the normal work or when shorted or a component is damaged. Hence, intrinsically safe design of the circuit is a kind of low power design; the current and voltage in the circuit will be strictly limited within an allowable range.

**Table 1:** States of the art in liquid-level sensors.

Sensor	Advantages	Disadvantages
Float liquid-level sensor	Simple structure	Vulnerability to mechanical damage
	Low cost	High maintenance cost
Ultrasonic liquid-level sensor	Simple structure	Susceptible to interferences
	Easy installation and maintenance	Difficult to achieve intrinsically safety performance
Magnetostrictive liquid-level sensor	High precision	Easy to get stuck in special environments such as the turbid liquid
	Large-scale	
	High security	
Differential pressure liquid-level sensor	Stable performance	Easily jammed or blocked
	High precision	
	Low cost	
Fiber-optic liquid-level sensor	Anti-electromagnetic interference	Unable to measure the turbid liquid level and other liquid with sticky substance
	Robustness toward harsh environments	
Conventional capacitive liquid-level sensor	Low cost	Complex structure
	High linearity	Non-intrinsic safety
	Low energy dissipation	

Based on such considerations, a novel, intrinsically safe liquid-level sensor system is designed and implemented to realize liquid level detection in flammable and explosive environments by using the poly vinyl chloride (PVC) coaxial cable. The fabrication of the sensors is straightforward and with a low cost. The paper is organized as follows: Section 2 presents the measuring mechanism and derivation of the theoretical values. In Section 3, a description of the experimental platform based on CAV444 is given, along with the protection system implementation and sparks safety assessment. Section 4 is devoted to testing the sensor prototype. The analysis results of the PVC performance are given to verify the theoretical calculation. Subsequently, a piecewise linearization approach is proposed to further improve the measuring accuracy. Finally, the conclusions are drawn in Section 5.

## 2. Measuring Mechanism

### 2.1. Sensing Element and Conversion Principle

**Figure 1:**
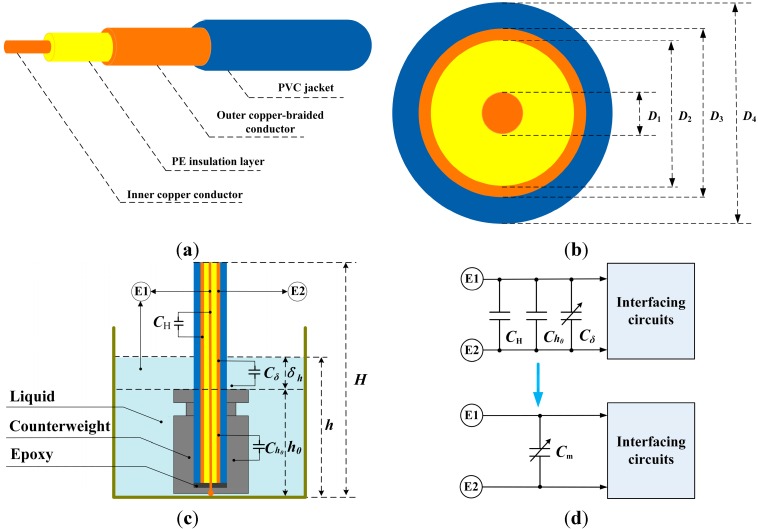
(**a**) Inner structure of poly vinyl chloride (PVC) coaxial cable; (**b**) Cross-sectional view of PVC coaxial cable; (**c**) Schematic view of liquid-level measurement; (**d**) Equivalent circuit of the measuring capacitance.

In the proposed intrinsically safe liquid-level sensor system, PVC coaxial cable is selected as the sensitive element. Due to the outstanding advantages of product capacity, material processability, thermal stability and cost efficiency, PVC has been one of the most widely used insulation material for wire and cable systems [19]. Furthermore, the inner structure and the cross-sectional view of the PVC coaxial cable are schematically shown in Figure 1a,b.

From Figure 1a, it can be observed that the PVC coaxial cable is composed of four layers from inside to outside: an inner copper conductor, a polyethylene (PE) insulation layer, an outer copper-braided conductor and a PVC jacket. Moreover, as depicted in Figure 1b, the diameters of the inner conductor, the PE insulation layer, the outer conductor, and the PVC jacket are, respectively, defined as *D*_1_, *D*_2_, *D*_3_ and *D*_4_*.*

In order to appropriately illustrate the measuring principle of the proposed sensor, two terminals (E1 and E2) are defined, as shown in Figure 1c,d. In principle, liquid level can be determined by measuring the capacitance between E1 and E2. As can be seen in Figure 1c, the inner copper conductor is connected to the liquid by a stainless counterweight. Moreover, a small amount of epoxy is injected into the counterweight to insulate the inner copper conductor from the outer copper-braided conductor. 

In the initial state, the capacitance between E1 and E2 is defined as *C*_0_. Obviously, *C*_0_ consists of two parts: *C*_H_ and *C*_h__0_. *C*_H_ is the capacitance between the inner copper conductor and the outer copper-braided conductor, and *C*_h__0_ is the capacitance between the outer copper-braided conductor and the counterweight. Then, as the liquid level varies from 0 to *h*_0_, the capacitance between E1 and E2 remains the same, which is always equal to *C*_0_. Afterwards, with further increase of the liquid level (>*h*_0_), the PVC coaxial cable is immersed deeper into the liquid and the capacitance between E1 and E2 will therefore increase. The capacitance increment, which is resulted from the liquid level increase, is defined as *C*_δ_. Consequently, the overall measuring capacitance *C*_m_ is equal to the sum of *C*_0_ and *C*_δ_. Corresponding equivalent circuit of the measuring capacitance is given in Figure 1d. Relevant calculating equations are as follows:

(1) $$C_{\text{H}}=2\text{π}{\text{ε}}_{0}{\text{ε}}_{r}H/\ln(D_{2}/D_{1}),C_{{\text{h}}_{0}}=2\pi {\text{ε}}_{0}{\text{ε}}_{t}h_{0}/\ln(D_{4}/D_{3}),C_{\text{δ}}=2\pi \varepsilon_{0}\varepsilon_{t}{\text{δ}}_{\text{h}}/\ln(D_{4}/D_{3})$$

(2) $$C_{0}\text{=}C_{H}+C_{h_{0}}=\text{2π}\varepsilon_{\text{0}}\varepsilon_{\text{r}}H\text{/ln (}D_{\text{2}}\text{ /}D_{\text{1}}\text{)}+\text{2π}\varepsilon_{\text{0}}\varepsilon_{\text{t}}h_{\text{0}}\text{/ln (}D_{\text{4}}\text{ /}D_{\text{3}}\text{)}$$

(3) $$C_{m}=C_{0}+C_{\text{δ}}=C_{0}+\text{2π}\varepsilon_{\text{0}}\varepsilon_{\text{t}}\delta_{\text{h}}\text{/ln (}D_{\text{4}}\text{ /}D_{\text{3}}\text{)}$$

where, ε_0_ is the vacuum electrical permittivity, ε_r_ is the relative electrical permittivity of the PE insulation layer, and ε_t_ is the relative electrical permittivity of the PVC jacket. H is the total length of the PVC cable, *h*_0_ is the height of the counterweight and δ_h_ is the distance between the liquid surface and top of the counterweight. From Equation (3), it can be observed that the overall measuring capacitance *C*_m_ and the liquid level change δ_h_ are an approximately linear relationship under ideal condition. Moreover, according to the relevant parameters indicated in Table 2, theoretical values *C*_m_ can be achieved.

**Table 2:** Relevant parameters.

Parameter	Value	Unit
ε_0_	8.85 × 10^−12^	F/m
ε_r_	2.3	Dimensionless
ε_t_	4.8	Dimensionless
*D*_1_	0.75	mm
*D*_2_	4.8	mm
*D*_3_	5.0	mm
*D*_4_	7.2	mm
*H*	3.0	m
*h*_0_	0.1	m
*h*	0.1~1.1	m
δ_h_	0~1	m
*C*_0_	279.89	pF

Hence, the theoretical values of *C*_m_ can be derived by substituting the above parameters into Equation (3). That is:

(4) $$C_{m}=C_{0}+{\text{C}}_{\text{δ}}\text{=279.89}+\text{731.98}\delta_{\text{h}}\approx \text{279.89\textasciitilde{}1011.87pf}$$

The calculation results show that over the liquid-level measurement range of 1.0 m, the measuring capacitance range is about 279.89~1011.87 pF theoretically. Moreover, these capacitive values to be measured are in the pF range, which is allowable in intrinsic safety design. The experimental studies will be conducted and analyzed in detail later.

### 2.2. Liquid-Level Sensor System Composition 

**Figure 2:**
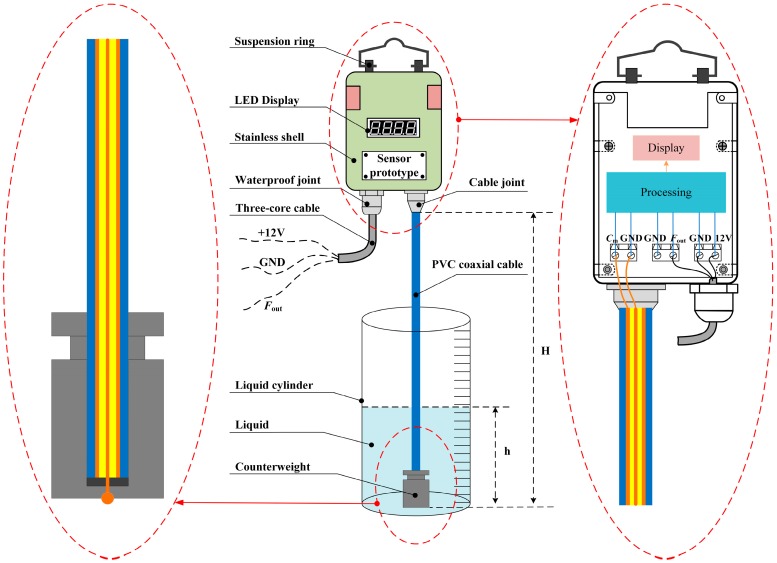
System composition of the designed liquid-level sensor.

Figure 2 illustrates the system composition of the designed liquid-level sensor. The capacitance of the PVC coaxial cable evolves with the length of cable submerged into the liquid, providing a mechanism to measure the liquid level. As can be easily seen, one end of the PVC coaxial cable (the inner conductor) is connected with the counterweight, while the other one is connected to the electrical circuits inside the stainless shell (150 × 105 × 48 mm) through a cable joint. Moreover, it can be seen that the inner copper conductor and the outer copper-braided conductor of the PVC coaxial cable are, respectively, connected to the terminals of GND and *C*_m_. Then, after a series of processing units, the capacitance signal is fed to the micro control unit (MCU), which controls the LED to display the liquid level and communicates with other monitoring devices through a transmitting frequency signal that contains the liquid level information. 

The suspension ring is utilized to hang the sensor prototype on fixtures. It is important to emphasize that all the electrical chips and circuits are placed inside the stainless steel shell with excellent sealing and anticorrosive properties. Furthermore, the system is designed with characteristics of intrinsic safety by limiting the energy of the circuit to avoid or restrain the spark and thermal effects. Both the power signal (12 V, GND) and the frequency signal (*F*_out_) are transmitted by the three-core cable through the waterproof joint. Moreover, the fabrication of the sensors is straightforward and with a low cost.

## 3. Sensor System Construction and Implementation

### 3.1. Signal Processing System 

**Figure 3:**
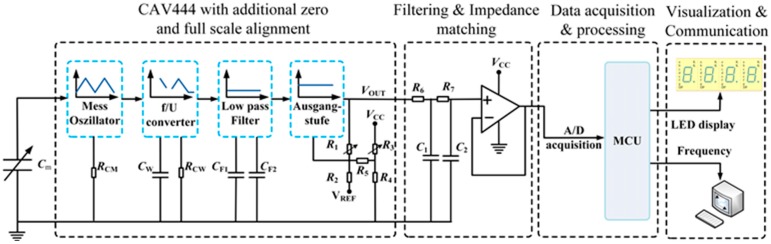
Overall block diagram of the signal processing system.

Figure 3 demonstrates the overall block diagram of the signal processing system. During the measurement, when the PVC coaxial cable is immersed in the liquid, the liquid level is linearly converted to the corresponding capacitance signal almost at the same time. Then, the measuring capacitance *C*_m_ is sent to CAV444 C/V converter chip.

As shown in the signal processing block diagram of CAV444 with additional zero and full-scale alignment, *C*_m_ works as the capacitor of the mess-oszillator built in CAV444. Furthermore, *C*_m_ is charged and discharged periodically with constant current of CAV444 and the period is a linear relationship with *C*_m_. Followed by a frequency/voltage converter and a low pass filter sequentially, an amplified DC voltage signal can ultimately be achieved at the output stage [20]. Afterwards, the obtained voltage signal is supplied to the filtering and impedance matching module to supply the MCU with enough signal energy. Finally, the measuring data are fed to the MCU, which control the LED to display the liquid level and communicate with other devices by a transmitting frequency signal that contains the liquid level information. 

Based on the advantages of the professional integrated circuit, a CAV444 C/V converter chip, which is produced by AMG Company, is adopted as the signal processor of the measuring capacitance. The transfer function of the CAV444 output signal *V*_OUT_ is given by [20]:

(5) $$V_{\text{OUT}}=A\cdot (3\cdot C_{\text{m}}\cdot \Delta V_{\text{CM}}\cdot R_{\text{CM}})/(8\cdot C_{\text{W}}\cdot R_{\text{CW}})+B\cdot V_{\text{REF}}$$

where, *C*_m_ is the measuring capacitance, Δ*V*_CM_ is defined by the internal resistors, and when *V*_CC_ = 5 V, it is a fixed voltage value (2.1 V). *C*_W_ is the capacitor of the frequency/voltage converter, and its value is connected with the value of *C*_m_, namely *C*_W_ = *C*_m(max)_/1.4. Resistors *R*_CM_ and *R*_CW_ are related to charge/discharge current and they can be calculated directly by the parameter optimization software (Kali_CAV444). The reference voltage *V*_REF_ is 2.5 V. *A* and *B* are both coefficients, which are only associated with the value of the resistors (*R*_2_, *R*_4_, *R*_5_, *R*_1_ and *R*_3_) [20].

where *R*_2_, *R*_4_ and *R*_5_ are fixed resistors, and *R*_1_ and *R*_3_ are adjustable resistors. Resistor *R*_1_ is adjusted for full scale, and *R*_3_ for zero scale; moreover, in the process of debugging, *R*_1_ and *R*_3_ are mutually correlated.

In order to obtain the optimal configuration of CAV444, the parameter optimization software (Kali_CAV444) is adopted for aligning the relevant parameters. Firstly, the theoretical values of *C*_m(min)_, *C*_m(max)_ and the expected values of *V*_DIFE(min)_ and *V*_DIFE(max)_ were input into the parameter optimization software. As mentioned in Section 2.1, the range for the theoretical value of *C*_m_ is from 279.89 pF to 1011.87 pF. Moreover, the range for the expected output voltage value of CAV444 (*V*_OUT_) is from 1.0 V to 2.3 V. Consequently, it is easy to achieve the value of *V*_DIFE_ by *V*_OUT_, namely that:

(6) $$V_{\text{DIFE(min)}}=V_{\text{OUT(min)}}-V_{\text{REF}}=\text{1.0}-\text{2.5}=-\text{1.5V; }V_{\text{DIFE(max)}}=\;V_{\text{OUT(max)}}-V_{\text{REF}}=\text{2.3}-\text{2.5}=-\text{0.2V}$$

The theoretical values and the expected values are presented in Table 3:

**Table 3:** Input parameters.

Parameter	Value	Unit
*C*_m(min)_	279.89	pF
*C*_m(max)_	1011.87	pF
*V*_DIFE(min)_	−1.5	V
*V*_DIFE(max)_	−0.2	V

Then, the preliminary calculation results are shown in Table 4.

**Table 4:** Preliminary calculation results.

Parameter	Value	Unit
*R*_CM_	125	kΩ
*C*_W_	1416.62	pF
*R*_CW_	125	kΩ
*R*_A_	60	kΩ
*C*_F1,F2(min)_	109.85	nF
*R*_1(meas)_	33	kΩ
*R*_3(meas)_	100	kΩ
*R*_2,_ *R*_4,_ *R*_5_	100	kΩ

Secondly, the output voltage of CAV444 was measured and the value of *V*_DIFE_ was recalculated as follows:

(7) $$V_{\text{DIFE(meas,min)}}=V_{\text{OUT(meas,min)}}-V_{\text{REF}}=\text{2.3}-\text{2.5}=-\text{0.2V; }V_{\text{DIFE(meas,max)}}=V_{\text{OUT(meas,max)}}-V_{\text{REF}}=\text{3.29}-\text{2.5}=\text{0.79V}$$

Afterwards, the modified values of *V*_DIFE_ _(min)_ and *V*_DIFE_ _(max)_ were input into the parameter optimization software again, as shown in Table 5.

**Table 5:** Input of measurements.

Parameter	Value	Unit
*V*_DIFE(meas,min)_	−0.2	V
*V*_DIFE(meas,max)_	0.79	V

The calculation results of the resistance are shown in Table 6.

**Table 6:** Calculation results of the resistance.

Parameter	Value	Unit
*R*_1(final)_	52.22	kΩ
*R*_3(final)_	1.8	kΩ

In summary, all the parameters mentioned above are determined by the parameter optimization software. According to the calculation results, the output voltage can be expressed as:

(8) $$V_{\text{OUT}}=1.13\times 10^{-3}C_{\text{m}}+1.26(V)$$

where, *C*_m_ is the measuring capacitance in pF and *V*_OUT_ is the output voltage of CAV444 in V. It can be seen that *V*_OUT_ is also an approximately linear function of *C*_m_ assuming ideal conditions. Moreover, as can been seen in Figure 3, after the process of second-order RC filtering, the voltage follower is adopted to meet the needs of impedance matching and improve the load capacity, *etc.*

### 3.2. Protection System and Sparks Safety Assessment 

**Figure 4:**
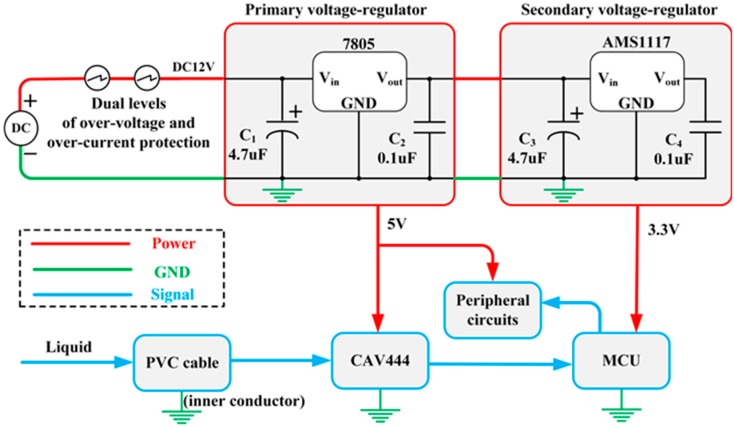
Schematic diagram of the power protection and conversion circuit system.

In the proposed liquid-level sensor system, the power protection and conversion circuit is designed elaborately. The power source is utilized to provide basic electrical power for the system. Then, dual levels of over-voltage and over-current protection circuit are designed to prevent electronic components from damage in cases of over-voltage or over-current conditions. Then, the voltage 12 V is sent to the primary voltage-regulator circuit, and the voltage is consequently dropped to 5 V. Afterwards, the 5 V voltage is fed to the secondary voltage-regulator circuit, through which the 3.3 V voltage can be achieved, which is the power supply for the MCU, while 5 V voltage for its peripheral circuits and CAV444. The schematic diagram of the power protection and conversion circuit system is illustrated in Figure 4.

#### 3.2.1. Dual Levels of Over-Voltage and Over-Current Protection

**Figure 5:**
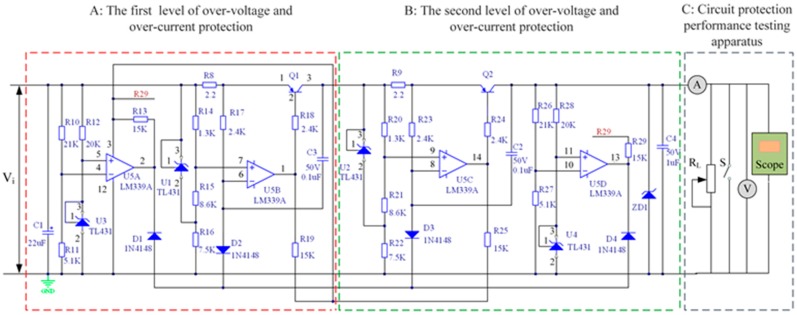
Dual levels of the over-voltage and over-current protection circuit and the apparatus of their protection performance test.

Figure 5 shows dual levels of the over-voltage and over-current protection circuit, which are intended to prevent electronic components from damage in cases of over-voltage or over-current. 

As can be observed, Figure 5 mainly consists of three parts: part A (the first level of over-voltage and over-current protection circuit), part B (the second level of over-voltage and over-current protection circuit), and part C (the apparatus for protection performance test). Except for some nuances, the functions of part A and part B are actual the same. When one of the two parts fails the protective effect, the other one can still work properly, thus the reliability of the circuit is improved. For simplicity, part A is utilized to illustrate the working principle of the protection circuit.

By analyzing the circuit of part A, it can be seen that Q1 is a key component, which controls the circuit in on or off state. Namely, in normal state, when the potential of pin 2 is lower than pin 1, Q1 is at an on state, thus the circuit can operate normally and the required output voltage or current can be achieved. However, if the potential of pin 2 is higher than pin 1, Q1 is at an off state, in which way the circuit can be protected. Further, the on-off state of Q1 is associated with U5A and U5B. 

As can be seen from part C, *R*_L_ is the load resistor. When the resistance of *R*_L_ is changed, the current flowing through R8 will change correspondingly. In a normal operating state, the potential of pin 6 of U5B is smaller than that of pin 7, and Q1 is at an on state. However, the potential of pin 6 will be larger than that of pin 7 as long as the load current exceeds a critical value. Consequently, Q1 will be at an off state and the circuit will be protected under over-current condition, with little output current. As can be seen from the circuit, the potential of pin 5 is fixed to 2.5 V under the control of the precision voltage regulator TL431. The value of the critical current *I*_c_ can be calculated from Equation (9):

(9) $$V_{i}-[\text{R14}/\text{(R14+R15})]\cdot 2.5=V_{i}-I_{c}\cdot \text{R8}$$

So from Equation (9), it can be calculated that *I_c_* is approximately 150 mA. Therefore, if the load current is larger than 150 mA, the circuit is in an off state, and thus is protected under over-current condition.

When the input voltage *V_i_* is within the normal range, the potential of pin 4 of U5A is lower than that of pin 5, so the potential of pin 6 of U5B is higher than that of pin 7 and Q1 is at an on state. However, the potential of pin 4 will be larger than that of pin 5 as long as the input voltage *V*_i_ exceeds a critical value. Consequently, the potential of pin 6 of U5B is lower than that of pin 7, and Q1 will be at an off state, in which way the circuit will be protected under over-voltage condition, with little output voltage. The value of the critical voltage *V_c_* can be calculated from Equation (10):

(10) $$[\text{R11}/\left(\text{R11+R10})\right]\cdot V_{c}=[5.1/\left(5.1+21\right)]\cdot V_{c}=2.5$$

So from Equation (10), it can be calculated that *V_c_* is approximately 12.5 V. Therefore, if the input voltage *V_i_* is larger than 12.5 V, the circuit is at an off state, and thus is protected under over-voltage condition.

**Figure 6:**
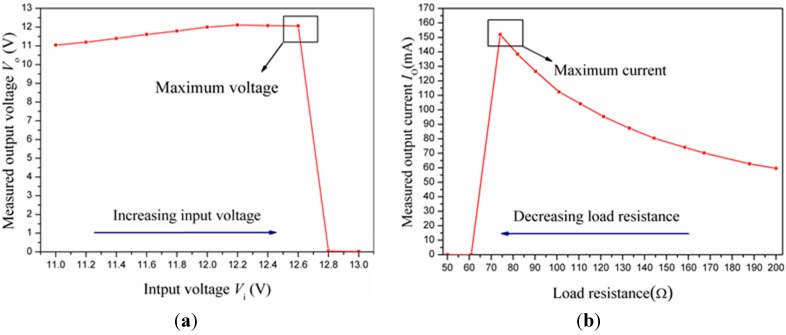
(**a**) Over-voltage protection curve and (**b**) over-current protection curve.

Part C in Figure 5 illustrates the apparatus of the protection performance test. We examined the function of over-voltage protection by boosting the input voltage from 11 V to 13 V with a 0.2 V step. From Figure 6a, the output voltage climbed slowly with the input voltage increasing from 11 V to 12 V. When the input voltage exceeded 12 V, it became roughly stable for a little while. Furthermore, if we increased input voltage to make it higher than 12.6 V, the measuring output voltage plummeted to nearly 0 V. Hence, the over-voltage protection was realized.

Similarly, we verified the function of over-current protection by reducing the load resistance R_L_ from 200 Ω to 50 Ω. The maximum output current of 152 mA was acquired in Figure 6b when load resistance was nearly 74 Ω. If we made load resistance less, the output voltage started decreasing, and finally dropped to 0 mA dramatically. Hence, the over-current protection was realized. The experimental curves of *V*_o_ *vs.* *V*_i_ and *I*_o_ *vs.* *R*_L_ are, respectively, presented in Figure 6a,b.

**Figure 7:**
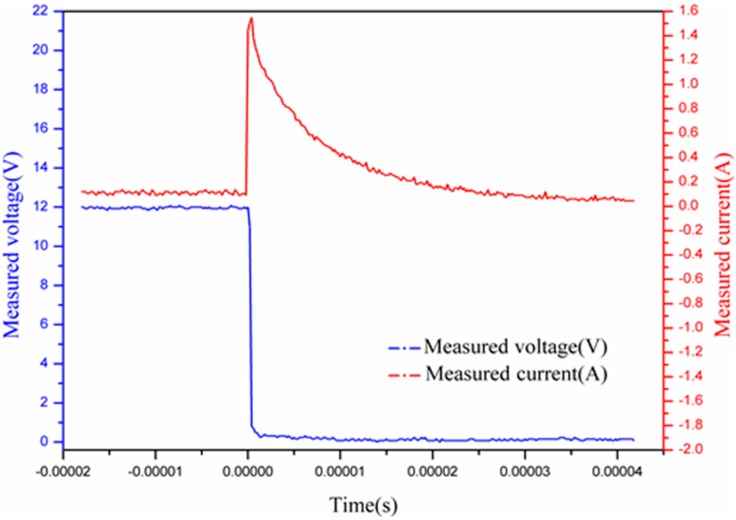
Short-circuit voltage and current protection curve.

As can obviously be seen, the circuit works normally when the switch S is disconnected. However, the output terminal of the protection circuit will be shorted when the switch S is closed. Consequently, a short-circuit test was carried out by controlling the switch S. The measured current and voltage are, respectively, presented in Figure 7.

As can be seen in Figure 7, the working current was about 100 mA and the voltage was about 12 V in the normal operating state. At the moment when the switch S was closed, the measured voltage reduced to 0 V within a short period of time (0.4 μS). Moreover, the measured current rose rapidly to 1.5 A in almost 0.3 μS, and then decreased to 0 A quickly (40 μS). Therefore, the conclusion that the circuit will be protected under short-circuit condition can be drawn.

#### 3.2.2. Sparks Safety Assessment 

To avoid or restrain the spark and thermal effects, all parameters of the circuit are designed in accordance with IEC 60079-0-2007 Explosive atmospheres—Part 0: Equipment-General requirements and IEC 60079-11-2006 Explosive atmospheres—Part 11: Equipment protection by intrinsic safety “i”. It is well known that the spark and thermal effects in circuit is very likely to occur and form a dangerous explosion source in flammable and explosive environments. Hence, the factor of inducing the spark and thermal effects must be considered seriously when the sensor system is designed.

First, the power supply assessment was carried out. As shown in Figure 4, the input voltage of the circuit is DC 12 V. In order to improve reliability of the circuit, the voltage should be multiplied by a safety factor *K* (1.5), that is:

(11) $$U^{\prime }=k\times U=1.5\times 12=18\text{V}$$

**Figure 8:**
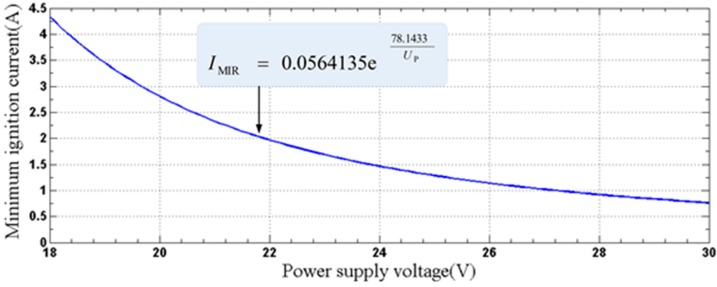
Resistive circuit spark ignition curve.

As mentioned in Section 3.2.1, the normal operating current is approximately 100 mA. When a short-circuit fault occurs in the output terminal of the protection circuit, the current reaches the maximum value (*I*_max_ = 1.5 A). The function of minimum ignition current (MIC) *I*_MIC_ and the power supply voltage *U*_P_ is expressed as [21]:

(12) $$I_{\text{MIC}}=0.0564135e^{\frac{78.1433}{U_{\text{P}}}}\text{A,18V}\leq {\text{U}}_{\text{P}}\leq \text{30V}$$

where the voltage range of the power supply is from 18 V to 30 V and Equation (12) is available in the environment at methane concentrations of from 8% to 8.6%. The corresponding resistive circuit ignition curve is presented in Figure 8.

According to Equation (12), the minimum ignition current *I*_MIC_ of the designed circuit is:

(13) $$I_{\text{MIC}}=0.0564135e^{\frac{78.1433}{U^{\prime }}}=0.0564135e^{\frac{78.1433}{18}}\approx 4.33(\text{A)}$$

Obviously, *I*_MIC_ is much larger than *I*_max_. From the perspective of spark ignition, the circuit is intrinsically safe.

Then, the capacitor assessment was also carried out. As can be seen in Figure 5, when a short-circuit fault occurs in the input terminal of the protection circuit, the energy of the capacitor (C1) reaches the maximum value. In the environment with the methane presence in a range of concentrations from 8% to 8.6%, the function of minimum ignition voltage (MIV) *U*_MIV_ and the capacitance value *C* is [21]:

(14) $$C=0.25579e^{\frac{103.263}{U_{\text{MIV}}}}\text{μF},(9V\leq U\leq 100V)$$

**Figure 9:**
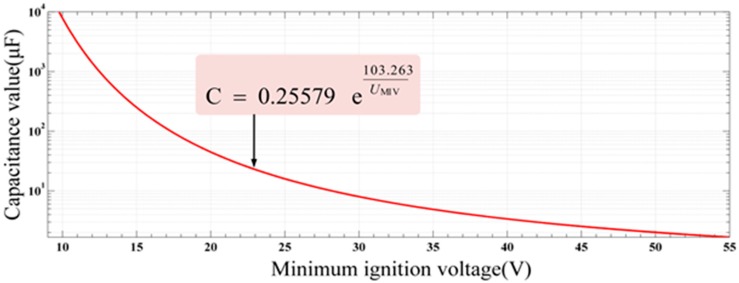
Capacitive circuit spark ignition curve.

The corresponding capacitive circuit ignition curve is shown in Figure 9.

According to Equation (14), the capacitance value that corresponds to the minimum ignition voltage value of 12 V can be calculated as follows:

(15) $$C=0.25579e^{\frac{103.263}{U^{\prime }}}=0.25579e^{\frac{103.263}{18}}\text{μF}\approx 79(\text{μF})$$

As shown in Figure 4 and Figure 5, the maximum capacitance value is 22 μF, which is lower than the calculated capacitive value *C*. Therefore, from the perspective of spark ignition, the above analyses and calculation results indicate that this intrinsically safe circuit is workable and reliable.

## 4. Experimental Demonstration and Discussion

### 4.1. Experimental Setup 

**Figure 10:**
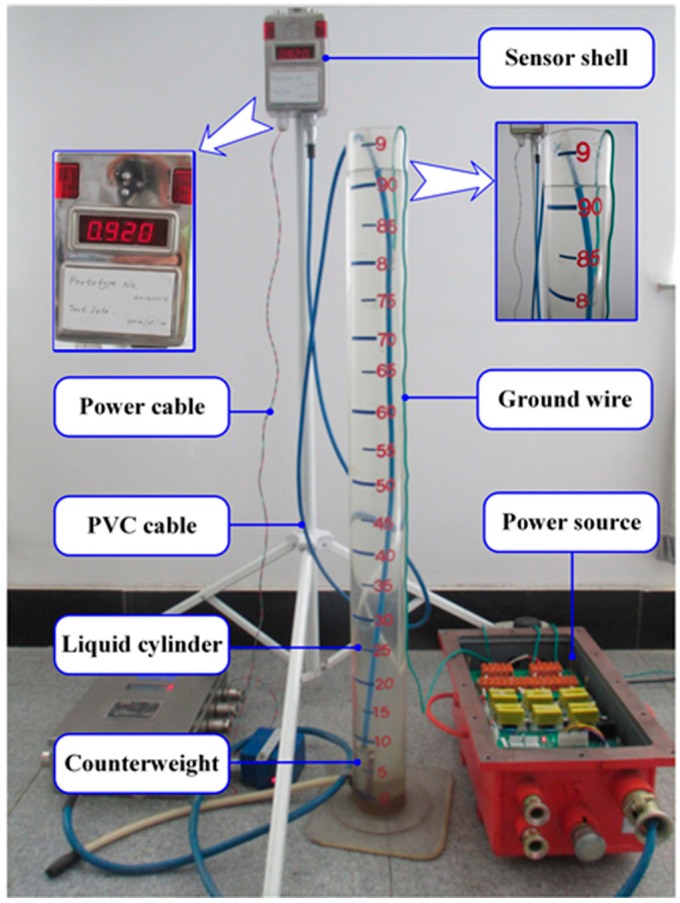
Experimental setup.

Figure 10 shows the experimental setup used to demonstrate the proposed sensor system. We used a bracket to fix the sensor prototype. The PVC coaxial cable with a counterweight was immerged into the liquid cylinder. The 12 V power source provided basic electrical power to the proposed sensor system through the power cable. In addition, it is important to mention that the measured liquid was connected to the earth by the ground wire with the purpose of imitating the real working condition of our designed sensor, because the sensor is commonly used to monitor the water level of a mine sump where the measured liquid is connected to the earth naturally but not stored in an insulating container. Here, the length of the PVC cable immerged into the measured liquid determines the liquid level.

### 4.2. Performance Test of the PVC Cable and the C/V Converter 

**Figure 11:**
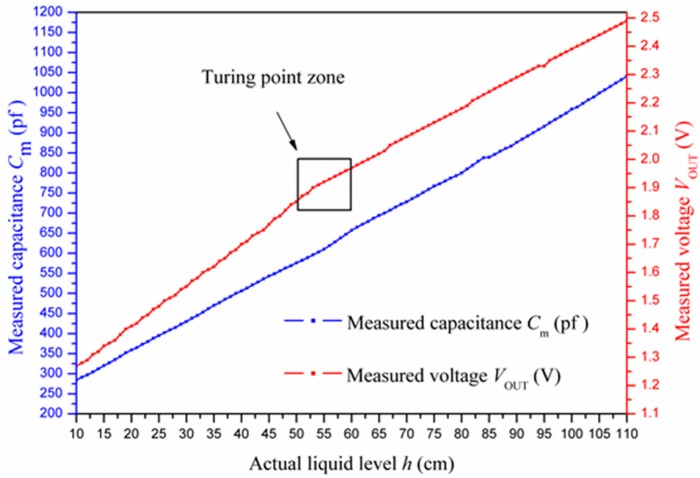
Experimental data of measured capacitance and voltage.

To evaluate the feasibility of the design, experiments were carried out to verify that the capacitance of the PVC cable and the output voltage of the C/V converter both altered approximately linearly with the change of the liquid level. After the experimental equipment shown in Figure 10 was prepared, the liquid (e.g., tap water) was added into the liquid cylinder slowly. The capacitance of the PVC cable (*C*_m_) and the output voltage of the C/V converter (*V*_OUT_) were measured and recorded with every one-centimeter incensement of the liquid level. Consequently, 100 sets of measured capacitance values and voltage values were obtained, respectively. For more accuracy, the same procedure was repeated three times to achieve the average values. Eventually, the values of *C*_m_ and *V*_OUT_ are plotted in Figure 11, as the liquid level rose from 10 centimeters to 110 centimeters.

From Figure 11, it was observed that the capacitance of the PVC coaxial cable increased from 284 pF to 1041 pF as the liquid level increased from 10 to 110 centimeters, and the experimental result was substantially consistent with the theoretical value (from 279.89 pF to 1011.87 pF) mentioned in Section 2.1. Meanwhile, the output voltage of the C/V converter increased from 1.27 V to 2.49 V, approximately in compliance with our theoretical voltage value (from 1.0 V to 2.3 V) as well. It could be concluded from the two curves that both the capacitance of the PVC coaxial cable and the output voltage of the C/V converter presented approximately linear relationships with the liquid level. However, we should also notice that the *V*_OUT_ curve had a turning point when the liquid level changed from 51 to 59 cm, which resulted from the intrinsic imperfection of C/V converter. We could eliminate this non-linear effects by piecewise linearization calibration described below.

### 4.3. Piecewise Linearization Calibration 

**Figure 12:**
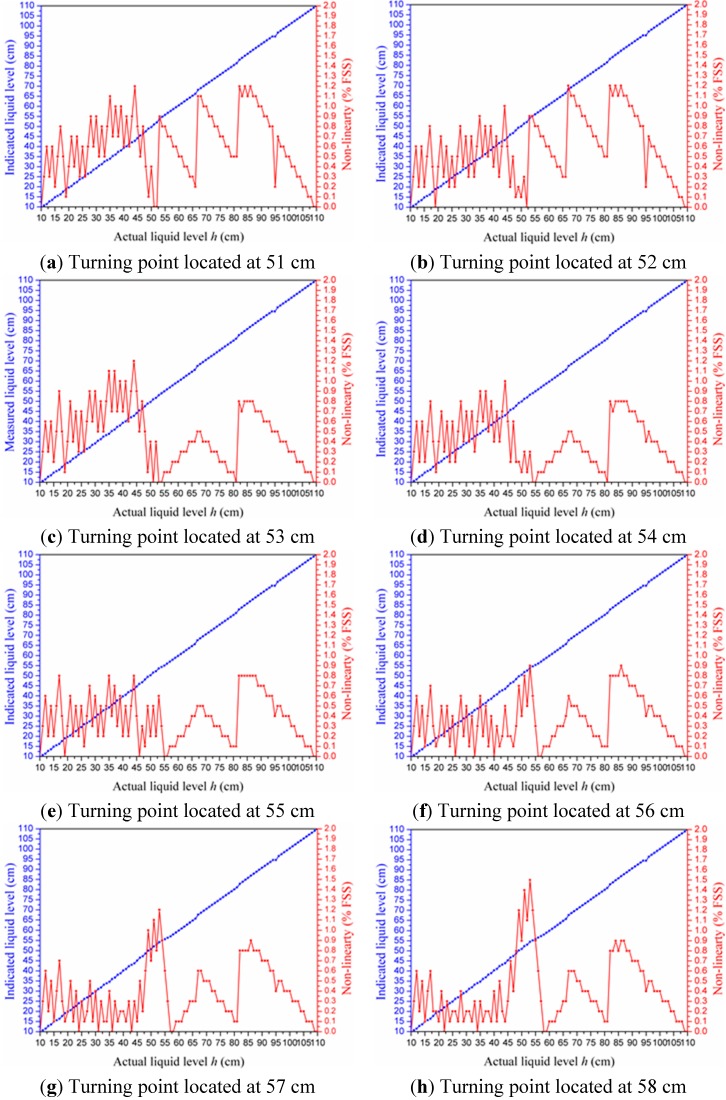

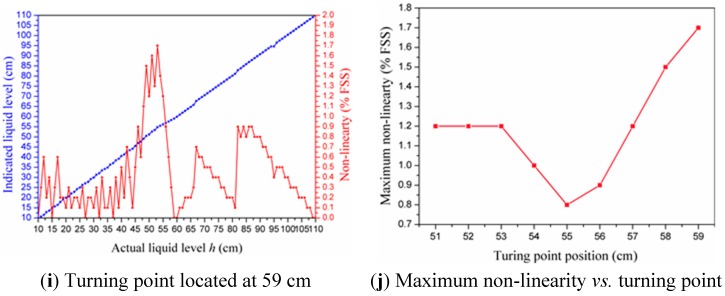
(**a**~**i**) Experimental data of the measured liquid level and non-linearity obtained when we selected, respectively, nine different turning points to develop a two-section piecewise linearization; and (**j**) maximum non-linearity *vs.* turning point position.

As mentioned in Section 4.2, the output voltage of the C/V converter had roughly linear relationships with the liquid level. Therefore, the liquid level could be calculated by developing a linearized approximation. Piecewise linearization was adopted to improve the accuracy. In order to locate an optimal turning point for piecewise linearization to attain the least non-linearity error, we successively selected nine integral points ranging from 51 cm to 59 cm as turning points to develop a two-section piecewise linearization, respectively. The experimental values of measured liquid level and non-linearity error are, correspondingly, given in Figure 12a~i.

We can conclude the fact from Figure 12 that which turning point we selected made a difference to the non-linearity of the sensor. The values of maximum non-linearity affected by the position of the turning point we selected are plotted in Figure 12j. Ultimately, the point of 55 cm was picked as the optimal turning point to develop a two-section piecewise linearization by which we can acquire the least non-linearity of 0.8 FSS %.

### 4.4. Resolution 

According to Equations (4), (8), (16) and (17) can be obtained:

(16) ΔC=731.98Δh

(17) $$\Delta V=1.13\times 10^{-3}\Delta C$$

where, Δ*h* is the liquid level change, Δ*C* is capacitance change caused by the liquid level change, and Δ*V* is the voltage change caused by the capacitance change. The resolution of the adopted A/D sampling chip is 12 bit and the reference voltage is 2.5 V. Thus, the minimum resolvable voltage is 0.61 mV, which approximately corresponds to 0.54 pF according to Equation (16). Then, the resolution of 0.74 mm can be derived from Equation (17). Such a resolution is quite acceptable for the liquid-level measurement in practice.

### 4.5. Repeatability 

**Figure 13:**
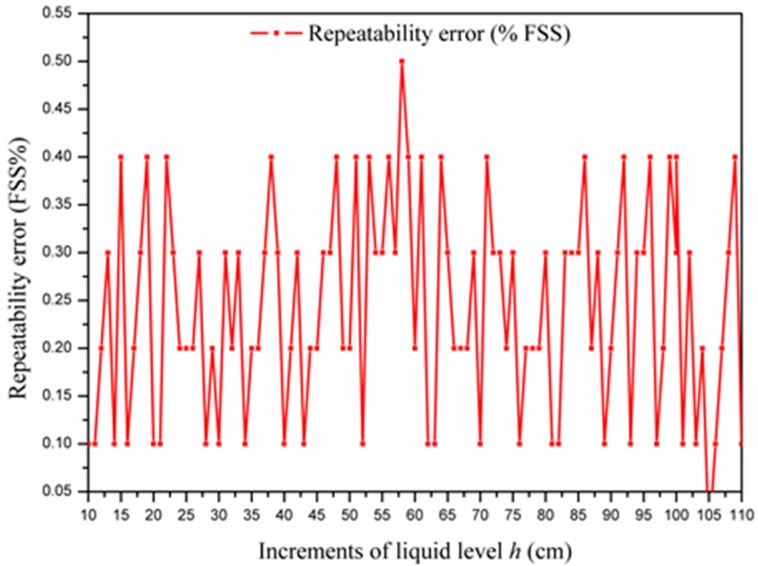
Experimental data of the repeatability test.

In the experiment, the repeatability of the sensor was also tested. At a fixed liquid level, pluralities of measurements are carried out repeatedly, and it was observed that there is a slight variation in the measurement result. The repeatability error is mainly resulted from the abrasion of the mechanical parts, auxiliary circuits drift and external parameters change. The experimental results of the repeatability error of the proposed liquid-level sensor are given in Figure 13.

As can be seen in Figure 13, the repeatability error varies with the increments of the liquid level. The maximum repeatability error (0.5 cm) occurs at 58 cm, while the minimum repeatability error (0 cm) at 105 cm. Therefore, the repeatability test shows that the proposed liquid-level sensor has excellent repeatability.

### 4.6. Hysteresis

**Figure 14:**
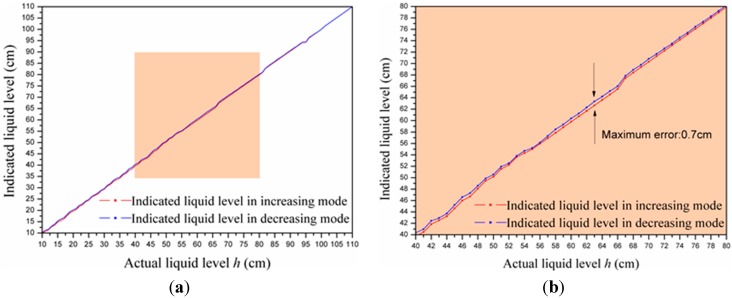
(**a**) Experimental data of the hysteresis test, and (**b**) and enlarged view of the shaded area in (a).

The liquid level was measured in increasing mode and then in decreasing mode, respectively, in order to evaluate the hysteresis effect of our proposed sensor. The testing process was repeated five times in total. We calculated the average value of every tested point in two different modes and plotted them in Figure 14. It can be seen that there exists a slight difference at the same level. When in the decreasing mode, the measured liquid values are a little higher than those in the increasing mode. From Figure 14, the maximum hysteresis error is 0.7% FSS, or 0.7 cm, when the actual liquid level is 63 cm. The reason for this hysteresis is the “flow-back phenomenon”: when the liquid level decreased, the water flow-back film was attached on electrode plate causing the hysteresis error [22].

**Figure 15:**
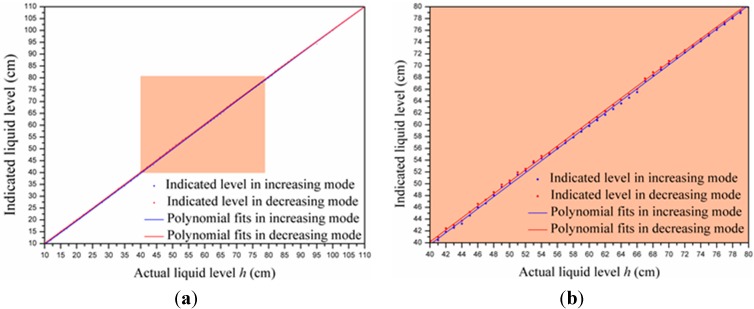
(**a**) Fitted results in increasing and decreasing mode; (**b**) and enlarged view of the shaded area in (a).

To avoid measurement error caused by the hysteresis effect, we took steps to realize hysteresis compensation. Figure 15 presents the fourth-order polynomial fits of indicated levels *vs.* actual levels. The two fitted results in increasing and decreasing mode are given by Equations (18) and (19), respectively.

(18) $$y_{1}=0.07837+0.9538x+1.28\times 10^{-3}x^{2}-1.005\times 10^{-5}x^{3}+1.951\times 10^{-8}x^{4}$$

(19) $$y_{1}=0.6545+0.9305x+2.21\times 10^{-3}x^{2}-2.275\times 10^{-5}x^{3}+7.218\times 10^{-8}x^{4}$$

**Figure 16:**
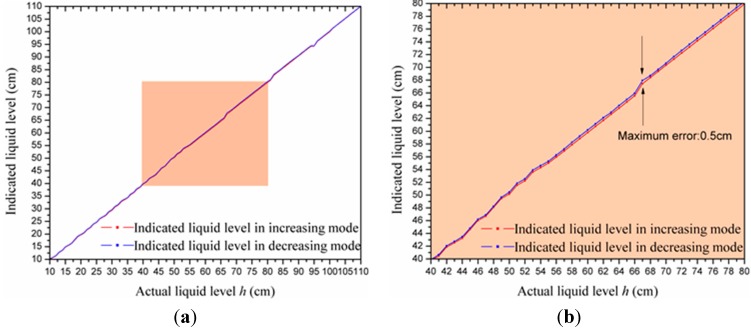
(**a**) Experimental data of the hysteresis test when applying software compensation algorithms; (**b**) and enlarged view of the shaded area in (a).

Software compensation algorithms can reduce the measurement error caused by the hysteresis effect, where Equation (18) is utilized to compensate hysteresis error when the actual level rises, while Equation (19) is exploited when the actual level declines. Then, the liquid level was measured again in increasing mode and then in decreasing mode, respectively. The testing process was also repeated five times. We calculated the average value of every tested point in two different modes and plotted them in Figure 16.

In Figure 16, it can be seen that the maximum hysteresis error is 0.5% FSS, or 0.5 cm, when the actual liquid level is 67 cm. Thus, the software compensation algorithms effectively reduce the hysteresis error.

In addition, the performance of the proposed liquid-level sensor is summarized in Table 7, including the resolution, measuring range, maximum nonlinearity error, maximum repeatability error, maximum hysteresis error, critical value of the over-voltage protection and over-current protection.

**Table 7:** Performance of the proposed liquid-level sensor.

Specification	Value	Unit
Resolution	0.74	mm
Measuring range	1.00	m
Maximum nonlinearity error	0.8	% FSS
Maximum repeatability error	0.5	% FSS
Maximum hysteresis error	0.5	% FSS
Critical value of over-voltage protection	12.6	V
Critical value of over-current protection	152	mA

## 5. Conclusions

A novel, intrinsically safe liquid-level sensor system utilizing a PVC coaxial cable is successfully designed and demonstrated. Measuring mechanism is analyzed and theoretical equations expounding the operation of the proposed sensor are derived. Then, the experimental platform of the liquid-level sensor system is constructed, which involves the entire process of measuring, converting, filtering, processing, visualizing and communicating. Additionally, the system is designed with characteristics of intrinsic safety by limiting the energy of the circuit to avoid, or restrain, sparks and thermal effects. Finally, by using the approach of piecewise linearization, a very good linearity is observed. The experiment results show that over the liquid-level measurement range of 1.00 m, the maximum nonlinearity error of the liquid-level sensor system is 0.8% FSS, the maximum repeatability error is 0.5% FSS and the maximum hysteresis error is 0.5% FSS. 

Compared to traditional capacitive sensors, the sensor innovation is the intrinsically safe design, which makes the proposed sensor suitable for liquid level detection in flammable and explosive environments. The current and voltage in the circuit are limited within an allowable range. Also, as the sensing element, the PVC coaxial cable is temperature-stable, non-porous, non-stick, flame retardant, and anti-corrosive. The experimental results show that the proposed sensor can meet the requirements of liquid-level detection in flammable and explosive environments. Furthermore, small size, low cost, easy fabrication and good environmental adaptability are further advantages of the proposed intrinsically safe liquid-level sensor.

However, more optimization in the design and fabrication can be made to create a truly perfect sensor system, such as the measurement range can be further enlarged; the size can be reduced even more, enabling the use in smaller applications; reduced power consumption; larger memory capacity; and adding new MCU features, such as infrared remote (IR) control, the auto calibration of the measuring system, *etc.*
